# Molecular Mechanisms of Chronic Intestinal Diseases in Response to Microbiota

**DOI:** 10.3390/cells11233722

**Published:** 2022-11-22

**Authors:** Laura Grasa

**Affiliations:** 1 Departamento de Farmacología, Fisiología y Medicina Legal y Forense, Facultad de Veterinaria, Universidad de Zaragoza, Miguel Servet, 177, 50013 Zaragoza, Spain; lgralo@unizar.es; 2 Instituto de Investigación Sanitaria de Aragón (IIS Aragón), 50009 Zaragoza, Spain; 3 Instituto Agroalimentario de Aragón -IA2-, Universidad de Zaragoza-CITA, 50013 Zaragoza, Spain

Chronic disorders of the intestine, such as inflammatory bowel diseases (IBDs) and irritable bowel syndrome (IBS), involve complex interactions between host and microbiota. This Special Issue has collected several studies showing the interaction between gut microbiota, the intestinal epithelial barrier and the intestinal immune system in the pathophysiology of these diseases.

It has been widely described that IBD patients show changes in microbiota composition and immune system dysregulation. Schierova et al. demonstrated that the changes in microbiota diversity observed in IBD patients treated with anti-TNF therapy depend on several factors such as disease severity, localization and surgical intervention. This longitudinal study also revealed that anti-TNF therapy increases the humoral response to multiple gut intestinal commensals [1].

Kriaa et al. explored the potential contribution of biliary acids (BAs) to intestinal inflammation and changes in gut microbiota observed in IBD. Bile acids play key roles in intestinal metabolism and cell signaling, and influence the gut microbial composition. In turn, the microbial metabolism of these BAs shape the host physiology. As alterations in BA metabolism and signaling influence intestinal homeostasis and drive gut dysbiosis in IBD, these molecules could be attractive therapeutic targets to treat this disease [2].

Although changes in the composition of the microbiota of IBD patients is well known, the role of the membrane vesicles (MVs) secreted by the gut bacteria remains unexplored. Kameli et al. described, for the first time, how bacterial MVs from Firmicutes and Proteobacteria can be important players in host–bacteria interactions. In this way, this study opens up a new field of study in the pathogenesis of this disease [3]. In this same context, Filip reviewed the role of extracellular vesicles secreted by different types of stem cells and intestinal bacteria, or that are present in fluids such as maternal milk, in the pathogenesis of IBD. These extracellular vesicles, which can contain different types of molecules such as proteins, RNA or DNA, can modulate the inflammation, the immune response and the integrity of the gut barrier. In addition, they can be useful as biomarkers or delivery vehicles for therapeutic applications [4].

Among the therapeutic strategies that are recently being proposed for the treatment of IBD is the modulation of the intestinal microbiota through probiotic bacteria. Tetragenococcus halophilus is a lactic-acid-producing bacterium that survives in environments with high salt concentrations and is involved in the fermentation process of soy sauce and fish sauce. Islam et al. evaluated whether T. halophilus has favorable outcomes against DSS-induced colitis in mice. The authors show that T. halophilus is effective in alleviating colitis in mice by altering immune regulation and gut microbiome composition. These results open up the possibility of using this bacterium as a complementary probiotic treatment to other therapies for ulcerative colitis [5].

Regarding the intestinal epithelial barrier, colonic epithelial cells are responsible for maintaining a delicate balance between luminal secretion and the absorption of fluids and ions. Deviations from homeostatic ion transport cause diarrhea, constipation and epithelial cell death and can contribute to IBD. Negussie et al. compiled an extended and elegant review about the model of colonic electrolyte secretion and absorption via the cystic fibrosis transmembrane regulator (CFTR), epithelial sodium channel (ENaC), Na-K-Cl cotransporters (NKCC1 and 2), Na-H exchangers (NHE1–4), colonic H, KATPase and several other key components involved in multilevel transepithelial ion transport. In fact, these colonic transport proteins have been evaluated as new targets for IBS constipation [6].

Epithelial barrier integrity is critical, as its dysfunction can lead to inflammation and the pathogenesis of immune disorders, such as IBD. Among the components of the intestinal barrier are resident macrophages and epithelial cells, which express adrenergic receptors and are receptive to norepinephrine, the primary neurotransmitter of the sympathetic nervous system (SNS). In their study, Mallesh et al. investigated the effect of SNS on mucosal immune and epithelial cell functions. They showed that a loss in sympathetic input induces a proinflammatory mucosal state, leading to reduced epithelial barrier function and enhanced antimicrobial defense. This implies that the SNS might be required to regulate intestinal immune homeostasis, epithelial function and microbial composition [7].

Recent epidemiological studies have shown a clear correlation between air pollution exposure and the increased hospitalization of patients with IBD. Phillippi et al. showed through a mouse model that exposure to inhaled diesel exhaust particles (DEPs), in conjunction with a high-fat diet, alters the intestinal epithelial barrier and evokes inflammation. The probiotic Winclove Ecologic^®^ seems to modulate the tight-junction proteins and inflammatory mediators to protect the intestine following exposure to inhaled DEPs [8].

IBS is a chronic, functional bowel disorder characterized by intestinal dysmotility. Changes in intestinal microbiota can lead to alterations in neuromuscular functions in the gut. In their study, Layunta et al. evaluated the role of TLR2 and TLR4, both receptors involved in the recognition of intestinal bacteria, in the intestinal motor-response induced by acetylcholine (ACh) in the mouse ileum. The authors showed, for the first time, that TLR2 acts on muscarinic M2 and M3 and nicotinic α3β4 ACh receptors, whereas TLR4 acts on muscarinic M3 and nicotinic α3β4 and α7 ACh receptors [9].

Finally, Goelz et al. presented a work on ascites, a condition that frequently accompanies other digestive pathologies such as gastrointestinal bleeding and bowel perforation. The authors used, for the first time, next-generation sequencing (NGS) methods to identify clinically relevant pathogens in ascetic fluid. These methods seem to be more effective than standard microbiological culture diagnostics, especially in detecting hard-to-culture anaerobic bacteria. This study demonstrated that patients with such infections might benefit from the use of NGS methods for the possibility of earlier and better targeted antimicrobial therapy, lowering the high mortality rates of these critically ill patients [10].
